# In Vivo Comparison of the Efficacy and Duration of Local Antibiotics on Smooth, Textured and Polyurethane Implant Surfaces

**DOI:** 10.1007/s00266-024-04090-2

**Published:** 2024-05-28

**Authors:** Ebubekir Karakas, M. Suhan Ayhan, Oguzhan Karasu, Ceren Ozkul Kocak, Meltem Yalinay

**Affiliations:** 1 Department of Plastic Reconstructive and Aesthetic Surgery, Faculty of Medicine, Gazi University, Ankara, Turkey; 2 Department of Pharmaceutical Microbiology, Faculty of Pharmacy, Hacettepe University, Ankara, Turkey; 3 Department of Medical Microbiology, Faculty of Medicine, Gazi University, Ankara, Turkey

**Keywords:** Breast implants, Polyurethane textured smooth, Biofilm, Antibiotics, BIA-ALCL, Bacteria, Microorganism

## Abstract

**Background:**

Capsular contracture is one of the most common complications after breast surgery involving silicone implants. The most likely cause of this condition is biofilm formation. In this study, the efficacy of local antibiotherapy against biofilm formation on implant surfaces was investigated.

**Methods:**

Thirty-six rats were divided into six groups. Three pockets were created on the dorsum of each rat, and 1 × 2 cm implant surface samples from smooth, polyurethane and textured implants were randomly placed into pockets. All samples were inoculated with staphylococcus epidermidis. In groups 1-2-3, inoculated samples were placed into the pockets and removed after 1, 6 and 24 h, respectively. In groups 4-5-6, inoculated samples immersed with rifamycin were placed and removed after 1, 6 and 24 h, respectively. Bacterial load was measured with plate count method.

**Results:**

Bacterial load was lower in groups 4-5-6 than in groups 1-2-3 (*p* < 0.05). In groups 4-5-6, bacterial load was lower for polyurethane than for textured surfaces at all time points (1, 6 and 24 h; *p *< 0.05). Again, in groups 4-5-6, bacterial load was lower for smooth than for textured surfaces at 24 h (*p *< 0.05). In groups 4-5-6, bacterial load was lower for polyurethane than for smooth surfaces at all time points, but difference was not statistically significant (1, 6 and 24 h; *p *< 0.05).

**Conclusion:**

The results suggest that local antibiotic therapy was effective in reducing the bacterial load on all surfaces. The effectiveness of local rifamycin on the polyurethane surface was higher, and the duration of activity was longer than other surfaces.

**No Level Assigned:**

This journal requires that authors assign a level of evidence to each article. For a full description of these evidence-based medicine ratings, please refer to the Table of contents or the online Instructions to Authors www.springer.com/00266.

## Introduction

### Background

Capsular contracture is one of the most common complications after breast augmentation or reconstruction with silicone implants [1–3]. One of the most common etiological factors leading to capsular contracture is chronic subclinical infection created by the biofilm layer [4–10]. The risk of breast-implant-associated anaplastic large cell lymphoma (BIA-ALCL), which has become a hot topic during the last decade, has also been shown to be related to the structure of the implant surface, biofilm formation on the implant surface and genetic predisposition [11–13].

Some in vivo animal studies have shown the strong relationship between biofilm formation and capsular contracture [14–17]. Therefore, it is recommended to rinse silicone implants with antibiotic solution prior to insertion into the surgical pocket [18, 19]. Furthermore, it has been demonstrated that the surface properties of the implants are important in the development of capsular contracture [20–25]. Previous long-term studies have reported that the rate of capsular contracture in polyurethane-coated implants was 15% less than those with textured surfaces and 30% less than those with smooth surfaces [26–28].

There are no studies in the literature comparing the effects of local antibiotic therapy on all three implant surfaces available in the market, which are smooth, textured and polyurethane surfaces. In our research, we planned to perform an in vivo animal study to measure the efficacy and duration of local antibiotic therapy, which is used to fight the formation of a biofilm layer, on implants with different surface properties.

### Objectives

*Hypothesis of the study:* Silicone implant surfaces have various physical properties, and microorganisms can remain attached to different surfaces at variable rates. Inserting silicone implants after rinsing with an antibiotic solution will reduce biofilm formation and thus the incidence of capsular contracture and BIA-ALCL. The holding capacity and duration of such an antibiotic solution and therefore the effectiveness of such antibiotics vary from surface to surface. Due to their hydrophilic nature and spongy structure, polyurethane-coated implants, which have higher liquid retention capacity as compared to silicone surfaces, will keep the antibiotic solution on their surfaces for a longer period. Therefore, the effectiveness on polyurethane surfaces should be higher as compared to implants with smooth and textured surfaces.

The main purpose of this study is to reveal the behavioral characteristics of different implant surfaces after bacterial contamination and the effectiveness of local antibiotic treatment after bacterial contamination at different timepoints in a standard experimental setting.

## Materials and Methods

### Experimental Animals

This study was carried out on 36 Wistar Albino female rats. Each weighed approximately 250 g. No other inclusion and exclusion criteria were applied.

### Study Design

All surgical procedures were performed by Karakas E, M.D. (EK) for all animals. A total of six groups, three experimental and three control groups consisting of six animals each, were created.

### Experimental Procedure

**Fig. 1:**
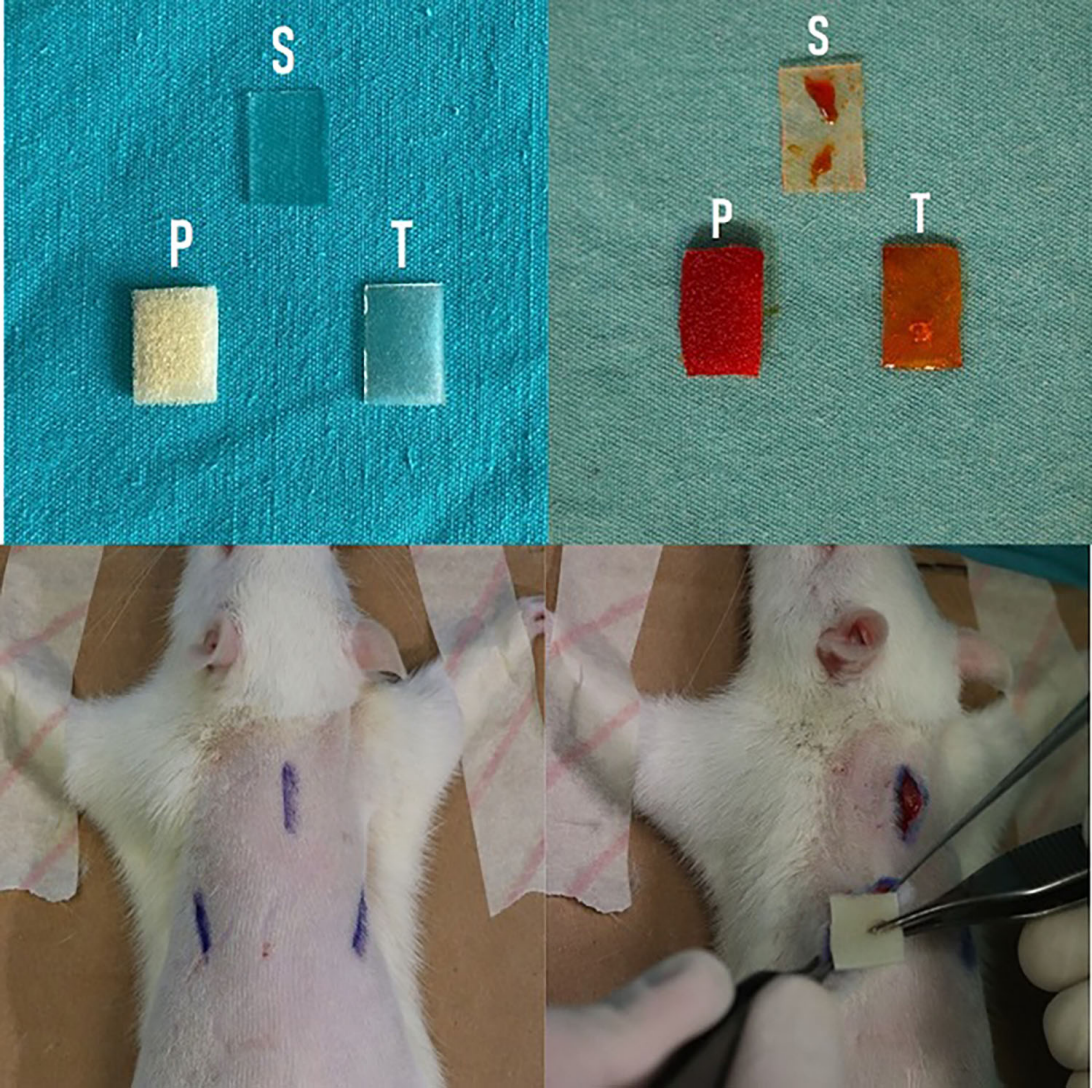
Smooth (S), polyurethane (P), textured (T) implant surface (without antibiotic) (Above left). Smooth (S), polyurethane (P) and textured (T) implant surfaces (with antibiotic) (Above right). Preoperative incision markings for submuscular pockets (Below left). Implant surface are placed in the submuscular pocket with sterile forceps (Below right)

The implant surface materials used in this study were obtained from three implants from two companies: the polyurethane implant surface (1 × 2 cm) was obtained from Microthane^®^ implants, manufactured by *Polytech Health and Aesthetics (Dieburg,Germany)*. Microthane^®^ is a polyurethane foam-coated implant surface with an average thickness of 1500 µ and a surface area of > 300 mm^2^. On the other hand, the textured implant surface (1 × 2 cm) was obtained from Siltex^®^ implants, and the smooth implant surface (1 × 2 cm) was obtained from the smooth implants manufactured by *Mentor Worldwide LLC (California, USA)* **(**Figure 1**)**. Siltex^®^ is a microtextured implant surface with a surface area of 100–200 mm^2^ that is textured using negative contact imprinting technique by pressing the uncured silicone mandrel into polyurethane foam. [28–31]

The three implant surface materials were placed on the dorsum of each rat under aseptic conditions. In all rats, a submuscular pocket was prepared using the same technique. After cleansing and covering, three different and disconnected pockets were prepared, one with a vertical incision of 1 cm from the nape and the other two with oblique incisions of 1 cm under both scapulae (Fig. 1). It was randomly decided which implant to be placed in which pocket in all animals. Randomization was done with a random number generating computer software.

In the control groups, the implant materials with three different surfaces were kept in a *Staphylococcus epidermidis* (*S. epidermidis*) culture (10^7^ CFU/ml) for 24 h, which has been clinically proven to form a biofilm layer. In the experimental groups, the implant materials with three different surfaces were first kept in an *S. epidermidis* culture (10^7^ CFU/ml) for 24 h and then inserted in a sterile solution containing 250 mg/3 ml of rifamycin for 5 min. Following the inoculation process, the implant surface materials were placed in the submuscular pockets, using sterile forceps (Fig. 1).

Each group included six rats, and the groups were composed as follows: Study groups are summarized in Table 1.

**Table 1:** Description of the groups

Groups	Implant surface	Bacteria	Antibiotic
Group 1 (1 h)	Smooth-Polyurethane-Textured	*Staphylococcus epidermidis*	–
Group 2 (6 h)	Smooth-Polyurethane-Textured	*Staphylococcus epidermidis*	–
Group 3 (24 h)	Smooth-Polyurethane-Textured	*Staphylococcus epidermidis*	–
Group 4 (1 h)	Smooth-Polyurethane-Textured	*Staphylococcus epidermidis*	Rifamycin
Group 5 (6 h)	Smooth-Polyurethane-Textured	*Staphylococcus epidermidis*	Rifamycin
Group 6 (24 h)	Smooth-Polyurethane-Textured	*Staphylococcus epidermidis*	Rifamycin

*Group* *1* *(Control group—1st* *h)*: Three different implant surfaces were placed in the submuscular pockets after inoculation with *S. epidermidis* and removed 1 h after insertion.

*Group* *2* *(Control* *group—6th* *h):* Three different implant surfaces were placed in the submuscular pockets after inoculation with *S. epidermidis* and removed 6 h after insertion.

*Group* *3* *(Control* *group—24th* *h):* Three different implant surfaces were placed in the submuscular pockets after inoculation with *S. epidermidis* and removed 24 h after insertion.

*Group* *4* *(Experimental* *group—Local antibiotic,* *1st* *h): *Three different implant surfaces were placed in the submuscular pockets after inoculation with *S. epidermidis*, kept in *rifamycin* solution for 5 min and removed 1 h after insertion.

*Group* *5* *(Experimental* *group—Local* *antibiotic,* *6th* *h):* Three different implant surfaces were placed in the submuscular pockets after inoculation with *S. epidermidis*, kept in *rifamycin* for 5 min and then removed 6 h after insertion.

*Group* *6* *(Experimental* *group—Local* *antibiotic,* *24th* *h): *Three different implant surfaces were placed in the submuscular pockets after inoculation with *S. epidermidis*, kept in *rifamycin* for 5 min and then removed 24 h after insertion.

### Preparation and Administration of Bacterial Inoculum

The biofilm-positive RP62A strain of *S. epidermidis* ([ATCC]35984; American Type Culture Collection, Manassas, VA, USA) was inoculated on Tryptic Soy Agar (TSA; Merck, Germany) medium and incubated at 37 °C for 24 h. After taking 4***–***5 colonies from single colonies with the help of a loop, these were inoculated into tryptic soy broth (TSB) medium and were incubated at 37 °C until the bacteria reached the logarithmic growth phase.

The bacterial culture in liquid medium was diluted, and a bacterial suspension was prepared in tryptic soy broth (TSB) corresponding to the 0.5 McFarland (10^8^ cfu/mL) standard. Then, the suspension was diluted to 1/10 to obtain a 10^7^ cfu/mL bacterial suspension. Sufficient bacterial inoculum (10^7^ cfu/mL, 5 mL) was added to the wells to cover the surface of the implants.

### Experimental Outcomes

Postoperatively, the animals were monitored in their own cages. During and after the surgical procedures, we did not observe any complications, such as bleeding or hematoma, in any of the animals. In the control groups, implant materials were removed from the pockets under sterile conditions after 1, 6 and 24 h in groups 1, 2 and 3, respectively. Similarly, in the experimental groups, implant materials were removed from the pockets under sterile conditions after 1, 6 and 24 h in groups 4, 5 and 6, respectively. As soon as the implants were removed, all rats were euthanized via drawing intracardiac blood.

### Microbiological Evaluation

The main outcome measure of this study was the number of bacteria on implant surfaces. To determine the number of bacteria on their surfaces, serial dilutions were made with PBS solution, and 100 microliters (μL) were inoculated into the TSA medium using the smear method to determine the number of viable bacteria. After being kept in an oven at 37 °C for 18 h, the colonies grown were counted. The number of bacteria per milliliter was calculated using the number of colonies grown in the media and the relevant dilution factor. This is the gold standard method for counting viable microorganisms. All microbiological evaluation was done by a blinded expert.

### Statistical Analysis

All data were analyzed using the IBM SPSS 15.0 *(SPSS Inc., Chicago, IL, USA)* package program. Descriptive statistics were expressed as median (minimum–maximum) for variables if the distribution was not normal. For the number of colonies, the significance of the difference in terms of median values was evaluated by using Mann–Whitney U and Kruskal–Wallis tests when the number of groups was two or more than two, respectively. Within the Kruskal–Wallis test, multiple comparisons were made using the post hoc Bonferroni test. The median numbers and mean ± standard deviation values of the colonies over time were evaluated with the Friedman test. This was done separately for surfaces with and without antibiotics and for different types of surfaces. Within the Friedman test, multiple comparisons were made using the Dunn–Bonferroni test. For *p *< 0.05, the results were considered statistically significant.

## Results

### Microbiological Evaluation

**Fig. 2:**
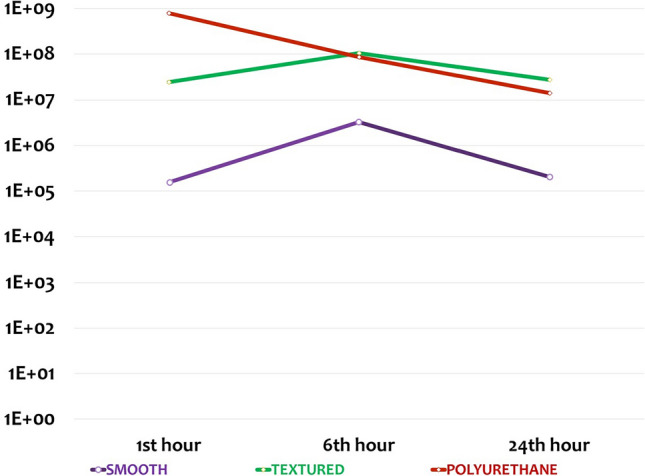
The mean number of bacterial colonies over time on surfaces to which local antibiotic treatment has not been applied

Bacterial load on each implant surface was measured quantitatively via scattering on the TSA medium. In groups without local antibiotic treatment, the least colonization was observed on smooth implant surfaces at all timepoints. In group 1 (1st h group without local antibiotic), the highest bacterial load was observed on polyurethane surfaces. At 6 and 24 h (Groups 2 and 3, respectively), the bacterial loads were equivalent on the polyurethane and textured surfaces; however, the smooth material remained the least populated surface (Fig. 2). The difference between the bacterial counts on smooth and other surfaces was statistically significant at all timepoints (Table 2).

**Table 2:** In groups without local antibiotic treatment (Group 1-2-3), bacterial load levels and statistical comparisons

Without local antibiotic treatment				
	Smooth	Polyurethane	Textured	*p*
	Median	Median	Median	
	Mean ± SD	Mean ± SD	Mean ± SD	
Group 1 (1 h)	130 k^**x**^ 156666,6 ± 78655,3^**b**^	145000 k^**y**^ 144166666,7 ± 60413298,7^**a**^	2350 k 5516666,6 ± 5869383,8	**< 0.001**
Group 2 (6 h)	2250 k^**x**^ 3296666,6 ± 2758272,4^**a**^	61000 k^**y**^ 87333333,3 ± 83595853,1	105000 k^**y**^ 107633333,3 ± 90804441,8	**< 0.05**
Group 3 (24 h)	145 k^**x**^ 206666,6 ± 226421,4^**b**^	14000 k^**y**^ 14683333,3 ± 6960004,7^**b**^	10500 k^**y**^ 28550000,0 ± 32285213,3	**< 0.01**
*p*	**< 0.01**	**< 0.01**	> 0.05	

The *p* values in bold are significant

Descriptive statistics were shown as medians (range between the quartiles) and mean ± standard deviation values.

After Kruskal–Wallis analysis, post hoc Bonferroni test performed on the rows and there was a significant difference between those with the letters **x** and **y** in their median values. After Friedman analysis, Dunn–Bbonferroni correction analysis was performed on the columns, and there was a significant difference between those with the letters **a** and **b** in mean ± standard deviation values.

k = 1000

**Fig. 3:**
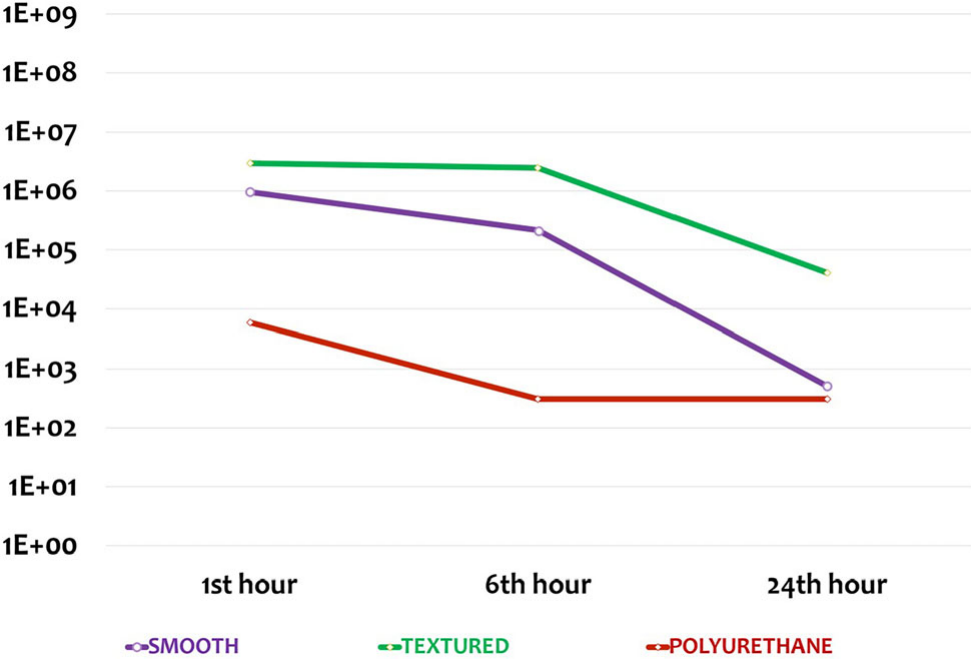
The mean number of bacterial colonies over time on surfaces to which local antibiotic treatment has been applied

In the experimental groups with local antibiotic treatment, the least colonization was observed on the polyurethane surface material at all timepoints. At 1 h and 6 h (Groups 4 and 5, respectively), the bacterial load on polyurethane surface material was found to be significantly lower than the colonization on the smooth and textured surface materials. However, at 24 h (Group 6), colonization levels on both the smooth and polyurethane surface were found to be significantly lower than on the textured surface (Fig. 3). Colonization was significantly higher at 1 h as compared to 24 h on all surface materials with antibiotics (Table 3).

**Table 3:** In groups with local antibiotic treatment (Group 4-5-6), bacterial load levels and statistical comparisons

With local antibiotic treatment				
	Smooth	Polyurethane	Textured	*p*
	Median	Median	Median	
	Mean ± SD	Mean ± SD	Mean ± SD	
Group 4 (1 h)	550 k 983333,3 ± 953764,4^**a**^	3000^**x**^ 4850,0 ± 5364,2^**a**^	2250 k^**y**^ 2995000,0 ± 2655663,7^**a**^	**< 0.01**
Group 5 (6 h)	255 k 217500,0 ± 146210,4	190^**x**^ 318,3 ± 397,2	2550 k^**y**^ 2550000,0 ± 2400188,8	**< 0.01**
Group 6 (24 h)	250^**x**^ 516,6 ± 775,6^**b**^	50^**x**^ 516,6 ± 825,6^**b**^	14500^**y**^ 42833,3 ± 77152,8^**b**^	**< 0.01**
*p*	**< 0.05**	**< 0.05**	**< 0.05**	

The *p* values in bold are significant

Descriptive statistics were shown as medians (range between the quartiles) and mean ± standard deviation values.

After Kruskal–Wallis analysis, post hoc Bonferroni test performed on the rows and there was a significant difference between those with the letters **x** and **y** in their median values. After Friedman analysis, Dunn–Bonferroni correction analysis was performed on the columns, and there was a significant difference between those with the letters **a** and **b** in mean ± standard deviation values.

k = 1000

In all groups, comparisons of the data for each surface material at different times were made separately using the Friedman test, and multiple comparisons were performed by using the Dunn–Bonferroni test. On the smooth surface without a local antibiotic, the highest number of colonies was measured at 6 h, and the lowest number was observed at 1 h; the difference was statistically significant. On the textured surface materials without a local antibiotic, the highest number and the lowest number of colonies were measured at 6 h and 1 h, respectively, but the difference was not statistically significant. On the polyurethane surface without a local antibiotic, the highest number and the lowest number of colonies were measured at 1 h and 24 h, respectively, and the difference was statistically significant (Table 2). On all surfaces treated with local antibiotics (Groups 4, 5 and 6), the highest number of colonies was measured at 1 h and, and the lowest number of colonies was measured at 24 h; the difference was statistically significant (Table 3).

For each surface material and time, a comparison of the data based on whether antibiotic treatment was performed, that is, between Groups 4, 5 and 6 and Groups 1, 2 and 3, was performed by using a Mann–Whitney U test. As compared to the untreated groups, bacterial colonization was significantly lower in the groups in which smooth and textured surfaces were treated with local antibiotics at 6 and 24 h. Bacterial colonization levels on the polyurethane surface at 1, 6 and 24 h were significantly lower in groups treated with local antibiotics than in untreated groups (Table 4).

**Table 4:** Comparison of groups without local antibiotics and with local antibiotics according to hours

	Without antibiotic (Median)	With antibiotic (Median)	*p* value
Smooth			
1th h	130 k	550 k	***p ***> 0.05
6th h	2250 k	255 k	***p *< 0.05**
24th h	145 k	250	***p *< 0.01**
Polyurethane			
1st h	145000 k	3000	***p *< 0.01**
6th h	61000 k	190	***p *< 0.01**
24th h	14000 k	50	***p *< 0.01**
Textured			
1st h	2350 k	2250 k	*p *< 0.05
6th h	105000 k	2550 k	***p *< 0.05**
24th h	10500 k	14500	***p *< 0.05**

Descriptive statistics were shown as medians (range between the quartiles).

The Mann–Whitney U test was performed on the row and median values were observed and *p *< 0.05 statistical significance.

k = 1000

### Outcomes and Estimation

From a clinical point of view, we have observed that smooth implant surfaces have shown the least adherence on the part of microorganisms in untreated cases, whereas polyurethane and textured surfaces have demonstrated more colonization than smooth surfaces. However, in cases involving local antibiotic treatment, the colonization around smooth and textured surface materials remained similar to untreated cases, but polyurethane surfaces demonstrated a significant decrease in bacterial load as compared to untreated polyurethane surfaces and treated smooth and textured surfaces at all timepoints.

Regarding the interaction between the surface and the microorganisms, these findings suggest that textured implants are the most disadvantageous implants among the three surfaces available in the market because these surfaces displayed the greatest number of colonies in both untreated and treated cases. In contrast, smooth implants have the least potential to be negatively affected by the presence of bacterial contamination because they exhibit the least adherence to microorganisms. Still, due to the limited efficiency of local antibiotics on smooth surfaces, colonization not significantly reduced and, thus, may still be a risk factor for capsular contracture. Polyurethane implants demonstrated the most impressive results after local antibiotic treatment because the number of bacterial colonies was significantly reduced in treated cases. This is a huge advantage in terms of eliminating the impact of bacterial contamination on breast implant surfaces.

### Adverse Events

There were no adverse effects in any of the control and experimental groups.

## Discussion

Capsular contracture is the most common complication after breast augmentation, and it may cause reoperations [1–3]. Additionally, breast-implant-associated anaplastic large cell lymphoma (BIA-ALCL), which may emerge between the capsule and the implant, has also given rise to doubts about silicone breast implants in recent years. The biofilm which is a syntrophic community of microorganisms in which cells stick to each other and often also to a surface in an extracellular polymeric matrix is shown to be a source of subclinical infection and the subclinical infection created by biofilm is still considered among the leading etiological factors in the development of capsular contracture. Also, biofilm and subclinical infection are among the most likely causes of BIA-ALCL. *Staphylococcus epidermidis (S. epidermidis)* is the most frequently isolated microorganism from capsular samples. This bacterium is a natural member of breast flora, while other members of the flora include *Propionibacterium acnes, Corynebacterium sp., Staphylococcus lugdunensis and hominis, Mycobacterias and others.* [7–9, 11–13, 32, 33]

In our study, we aimed to evaluate the efficacy and duration of local antibiotic therapy on implants with different surfaces in terms of preventing the formation of a biofilm layer and related subclinical infection. The bacterial load on the removed implant surfaces was determined by colony counting using mediums, which is the gold standard method.

There are effective irrigation procedures described for local antibiotic treatment around the implants, such as povidone-iodine (betadine), bacitracin+sefazolin+gentamicin (Adam’s triple), betadine+sefuroxim+gentamicin (Giardano’s triple), betadine+saline and other combinations. Irrigation with these procedures has been shown to significantly reduce capsular contracture. The US Food and Drug Administration (FDA) banned the use of betadine in the spring of 2000, on the grounds that it could cause spontaneous implant rupture but FDA removed the ban against betadine use for breast implant in 2017. Authors such as Wiener suggest that there is no evidence betadin use causes spontaneous implant rupture [34–39]. In this study, we preferred rifamycin, as a single variable, due to its wide spectrum of action on both Gram (**+**) and Gram (-) bacteria and efficacy in preventing the atypical mycobacterial infection and the subclinical inflammatory process.

**Fig. 4:**
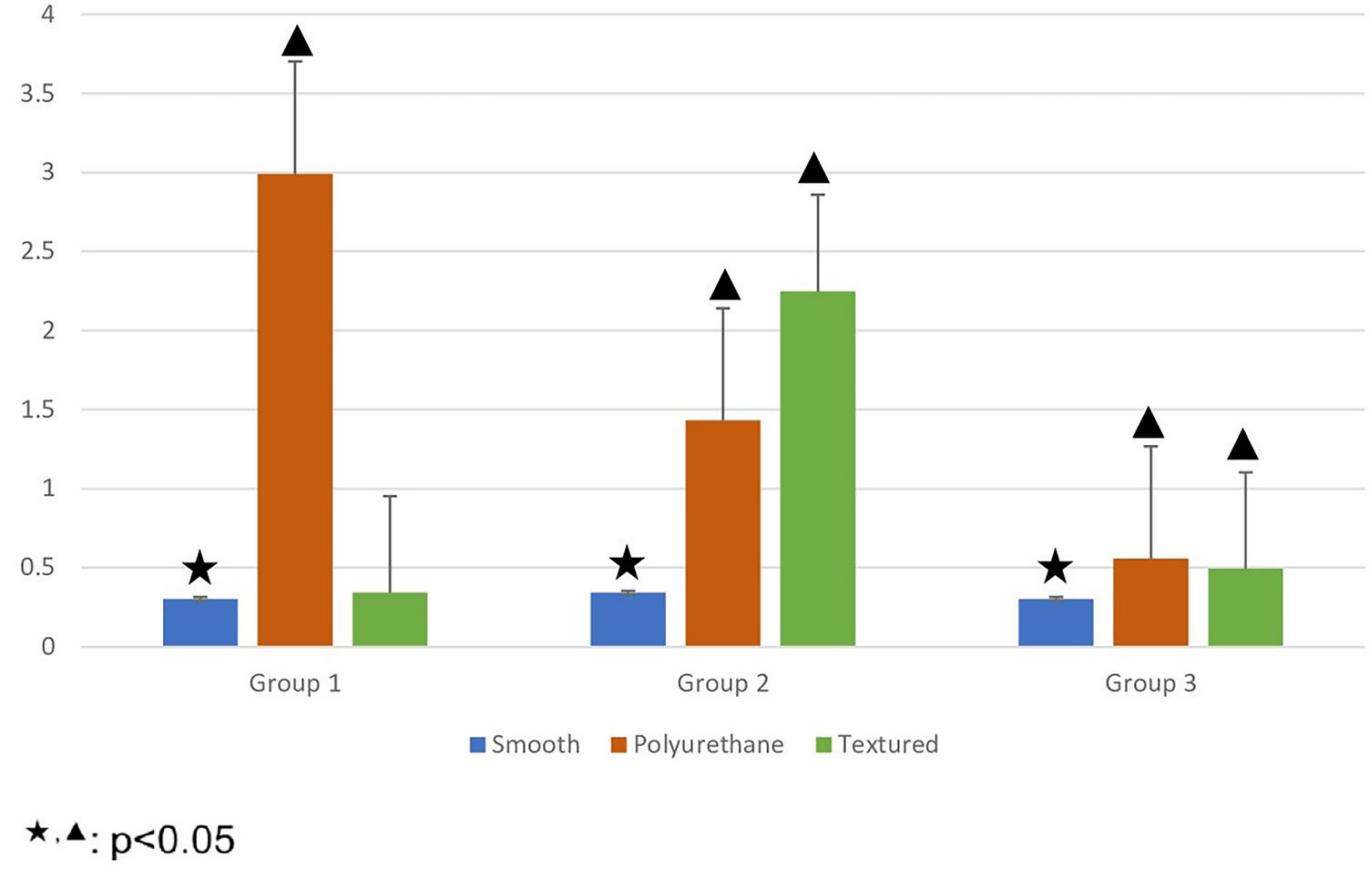
Graph showing statistical comparisons with error bars, without local antibiotic therapy (Groups 1-2-3). Small values could not be shown in the graph due to large differences between the data. Therefore, the data were made suitable for display by using the standardization method

In the groups without local antibiotic treatment, the bacterial loads on the polyurethane and textured surfaces were measured above the standard value (10^7^) at all timepoints. In these groups, the bacterial load on smooth surfaces was below the standard value (10^7^) but increased over time (Figure 2). In addition, significant differences were found between the smooth and polyurethane surfaces at all timepoints, as well as between the smooth and textured surfaces at 6 and 24 h, with the smooth surface indicating better results. However, there was no significant difference between polyurethane and textured surfaces at any timepoints (Figure 4).

**Fig. 5:**
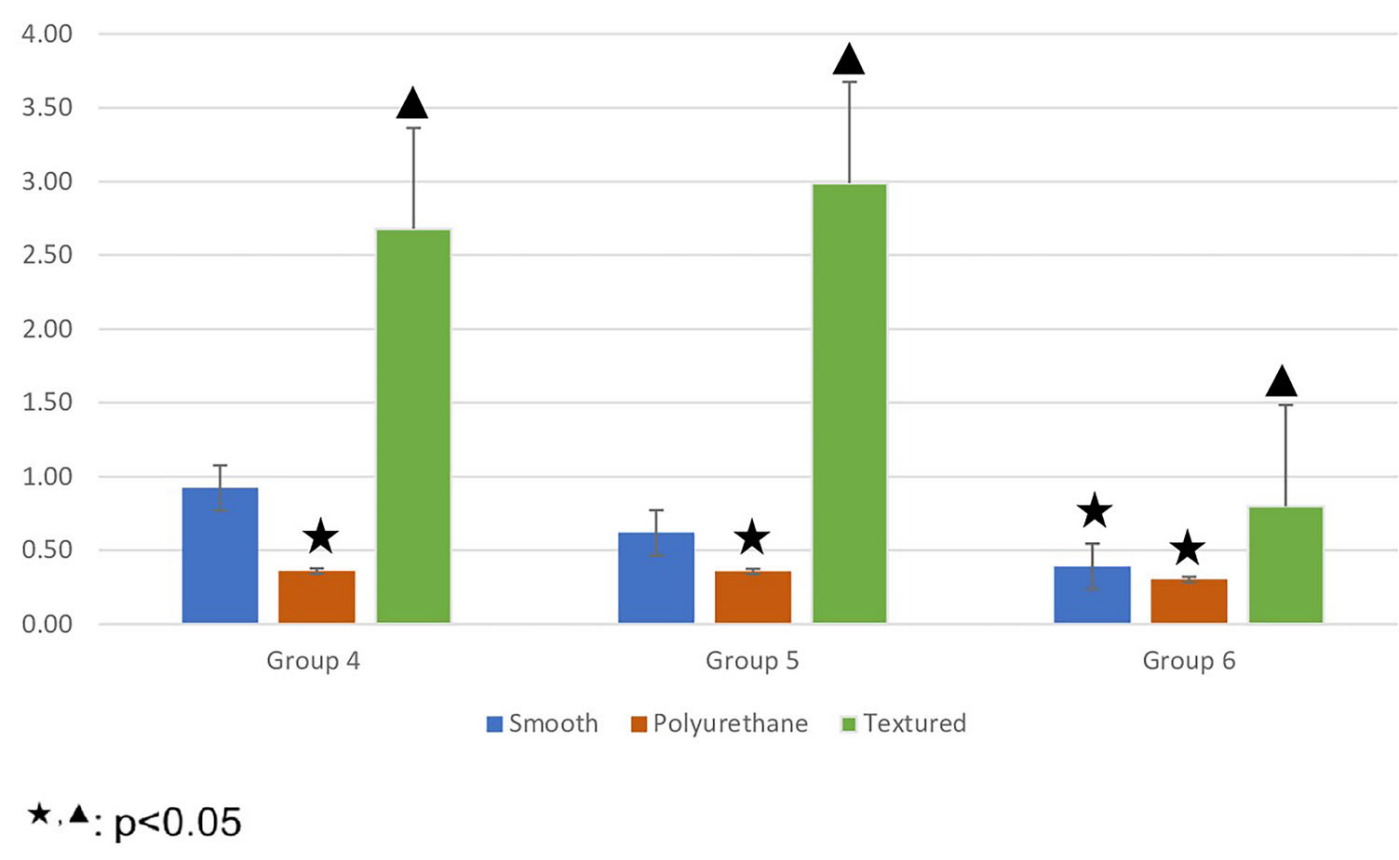
Graph showing statistical comparisons with error bars, without local antibiotic therapy (Groups 4-5-6). Small values could not be shown in the graph due to large differences between the data. Therefore, the data were made suitable for display by using the standardization method

On the other hand, bacterial loads on all surfaces were measured below the standard value (10^7^) in all groups with local antibiotic treatment. A dramatic decrease in bacterial load was observed on polyurethane surfaces. In these groups, there was a significant difference between polyurethane and textured surfaces, one in favor of polyurethane surface, at all timepoints. In addition, a significant difference was observed between the smooth and textured surfaces, one in favor of smooth surfaces, at 24 h. However, the difference between the polyurethane and smooth surfaces was not significant, even though there was less colony load on the polyurethane surface (Figure 5).

**Fig. 6:**
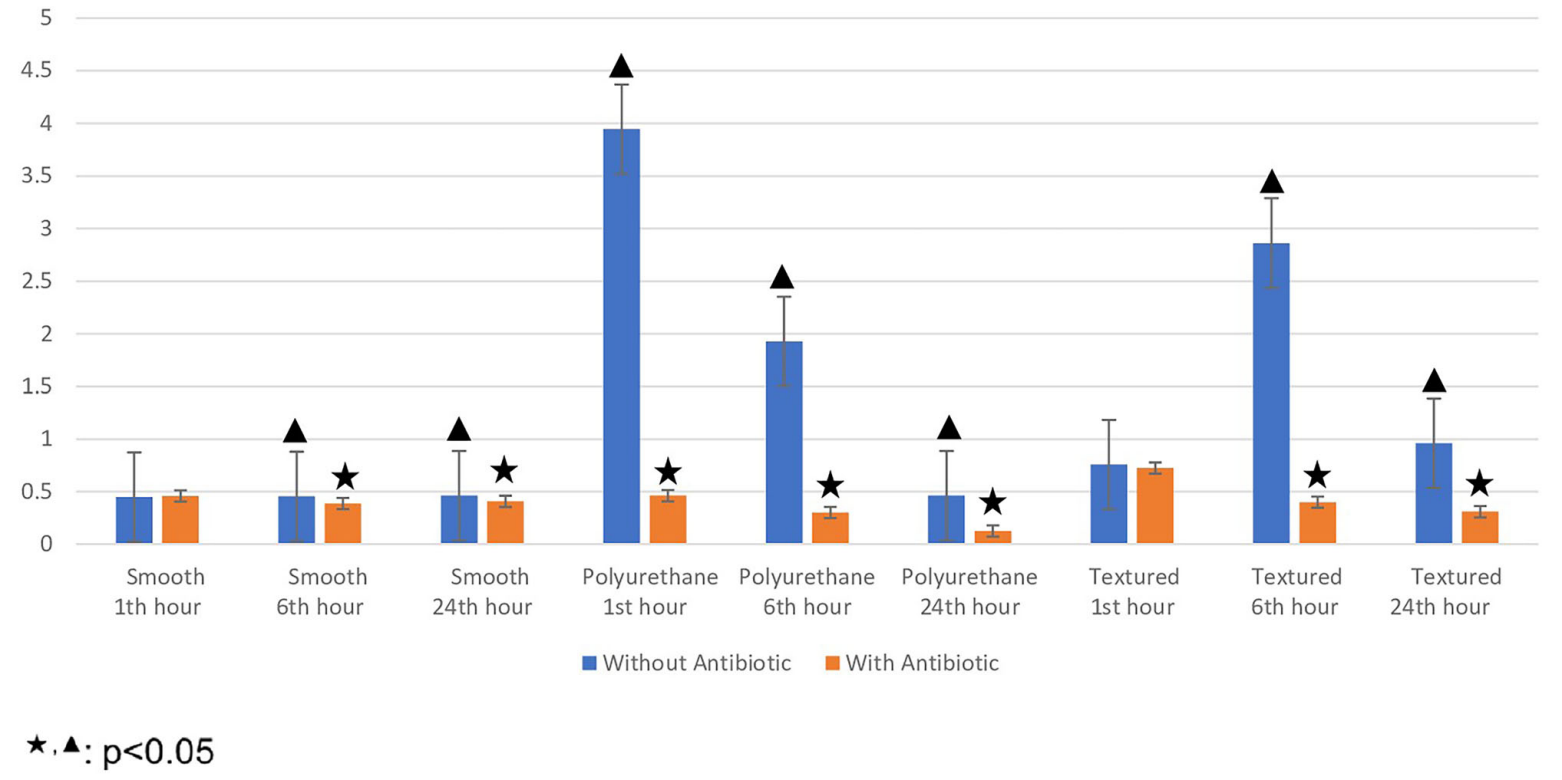
Graph showing statistical comparisons of groups without local antibiotics and with local antibiotics according to hours with error bars. Small values could not be shown in the graph due to large differences between the data. Therefore, the data were made suitable for display by using the standardization method

When the surface materials with and without antibiotic treatment (Groups 4, 5 and 6 versus Groups 1, 2 and 3) are compared; at 1 h, the difference was not significant between the smooth surfaces with and without antibiotics; however, a significant difference was observed in favor of the antibiotic-treated samples at 6 and 24 h on smooth surfaces. At 1 h, the difference between the textured surfaces with and without antibiotics was not significant; however, a significant difference was observed, one in favor of antibiotic-treated samples, at 6 and 24 h. On polyurethane surfaces, the difference was significant at all times and in favor of antibiotic-treated samples (Figure 6).

The bacterial load on all surfaces was significantly decreased at 24 h as compared to 1 h, even it was zero on half of the polyurethane and smooth samples at the 24 h. We can conclude that, although the bacterial load on the polyurethane surface was higher than that on the smooth surface at the beginning, local antibiotic treatment maintained its presence and effectiveness on the surface for 24 h and reduced the bacterial load regularly. On smooth surfaces, the bacterial load likely did not increase over time, due to the weak adhesion of the bacteria to the smooth surfaces from the beginning, and the load was decreased by the effect of antibiotics.

There was a significant decrease in bacterial colonization on polyurethane surfaces from the first h, while on smooth and textured surfaces, this occurred at 6 and 24 h, respectively. This may indicate the effectiveness of antibiotics on polyurethane from the 1st h, earlier than for smooth and textured surfaces (Figs. 2, 3**).** The higher duration and strength of local antibiotic treatment on polyurethane surfaces can be attributed to their superior absorption capacity as compared to other surfaces due to the spongy and hydrophilic structure of this material.

In samples without local antibiotics, we detected the lowest bacterial colony count on smooth surfaces, and this count remained stable over 24 h. However, on textured and polyurethane surfaces, bacterial load was higher than on smooth surfaces and, similarly, remained stable over 24 h. On the other hand, in samples with local antibiotics, we observed a significant decrease in bacterial colonies on polyurethane surfaces within 24 h, beginning with the 1st h. On smooth and textured surfaces with antibiotics, the decrease in bacterial load started with the 6th h, and the bacterial load on smooth surfaces was similar with those on polyurethane surfaces at the 24th h. However, the bacterial load on the textured surfaces was found to be higher than that on the other two surfaces at the 24th h.

Limitations of this study include lack of in vitro experiments, studying with only staphylococcus epidermidis and rifamycin. If there were more variables, more valuable findings could be obtained.

## Conclusion

Although the initial bacterial load on polyurethane surfaces was higher as compared to other two surfaces, the effect of antibiotics began more quickly on polyurethane surfaces than smooth and textured surfaces and effectively reduced the bacterial load within 24 h. This may be attributed to the fact that polyurethane surface is spongy and more hydrophilic than the other materials. Bacterial colonization on smooth surfaces was also lower than that on textured and polyurethane surfaces from the beginning of the experiment, and bacterial load was significantly reduced with local antibiotic treatment within 24 h. However, on textured surfaces, we observed that the bacterial load was higher than that on smooth surfaces, which similar to the results for polyurethane surfaces, from the beginning of the experiment, but the local antibiotic treatment did not respond as effectively as on the polyurethane and smooth surfaces. Ultimately, the number of bacterial colonies on the textured surfaces at the 24^th^ h was higher than on the other two surfaces, despite local antibiotic treatment.

This pioneering study comparing three different surface materials has shown that surface materials have different responses in the scenario of bacterial contamination. While smooth implants display the advantage of reduced bacterial adherence on the surface, textured implants remain the most inappropriate implants in terms of the bacterial load and following inflammatory response. On the other hand, polyurethane implants prove to be advantageous due to the fast and effective antibiotic response despite highest bacterial load among three surface materials.
